# Genetic evidence for contribution of human dispersal to the genetic diversity of EBA-175 in *Plasmodium falciparum*

**DOI:** 10.1186/s12936-015-0820-2

**Published:** 2015-08-01

**Authors:** Yoshiki Yasukochi, Izumi Naka, Jintana Patarapotikul, Hathairad Hananantachai, Jun Ohashi

**Affiliations:** Department of Biological Sciences, Graduate School of Science, The University of Tokyo, 7-3-1, Hongo, Bunkyo-ku, Tokyo, 113-0033 Japan; Department of Human Genetics, Graduate School of Medicine, The University of Tokyo, Tokyo, Japan; Department of Microbiology and Immunology, Faculty of Tropical Medicine, Mahidol University, Bangkok, Thailand; Department of Social and Environmental Medicine, Faculty of Tropical Medicine, Mahidol University, Bangkok, Thailand

**Keywords:** Diversifying selection, EBA-175, Human evolution, Out-of-Africa, *Plasmodium falciparum*

## Abstract

**Background:**

The 175-kDa erythrocyte binding antigen (EBA-175) of *Plasmodium falciparum* plays a crucial role in merozoite invasion into human erythrocytes. EBA-175 is believed to have been under diversifying selection; however, there have been no studies investigating the effect of dispersal of humans out of Africa on the genetic variation of EBA-175 in *P. falciparum*.

**Methods:**

The PCR-direct sequencing was performed for a part of the *eba*-*175* gene (regions II and III) using DNA samples obtained from Thai patients infected with *P. falciparum*. The divergence times for the *P. falciparum eba*-*175* alleles were estimated assuming that *P. falciparum/Plasmodium reichenowi* divergence occurred 6 million years ago (MYA). To examine the possibility of diversifying selection, nonsynonymous and synonymous substitution rates for *Plasmodium* species were also estimated.

**Results:**

A total of 32 *eba*-*175* alleles were identified from 131 Thai *P. falciparum* isolates. Their estimated divergence time was 0.13–0.14 MYA, before the exodus of humans from Africa. A phylogenetic tree for a large sequence dataset of *P. falciparumeba*-*175* alleles from across the world showed the presence of a basal Asian-specific cluster for all *P*. *falciparum* sequences. A markedly more nonsynonymous substitutions than synonymous substitutions in region II in *P. falciparum* was also detected, but not within *Plasmodium* species parasitizing African apes, suggesting that diversifying selection has acted specifically on *P. falciparumeba*-*175*.

**Conclusions:**

*Plasmodium falciparumeba*-*175* genetic diversity appeared to increase following the exodus of Asian ancestors from Africa. Diversifying selection may have played an important role in the diversification of *eba*-*175* allelic lineages. The present results suggest that the dispersals of humans out of Africa influenced significantly the molecular evolution of *P. falciparum* EBA-175.

**Electronic supplementary material:**

The online version of this article (doi:10.1186/s12936-015-0820-2) contains supplementary material, which is available to authorized users.

## Background

Approximately 200 *Plasmodium* species parasitize different vertebrate groups, including lizards, birds, and mammals [1]. Of them, five *Plasmodium* species, namely *Plasmodium falciparum*, *Plasmodium vivax*, *Plasmodium ovale* (consisting of two subspecies, *Plasmodium ovale curtisi* and *Plasmodium ovale wallikeri* [2]), *Plasmodium malariae*, and *Plasmodium knowlesi*, can infect humans. In particular, *P. falciparum* causes the most severe form of malaria in humans; however, when compared with *Plasmodium reichenowi* found in chimpanzees (*Pan troglodytes*), *P. falciparum* appears to be phylogenetically distantly related to other human malaria parasites [1, 3–5].

The 175-kDa erythrocyte binding antigen (EBA-175) of *P. falciparum* binds with a sialic acid on human glycophorin A (GYPA); the interaction of these molecules is the part of a major pathway for malaria parasite invasion into erythrocytes. The primary structure of *eba*-*175* gene is divided into seven regions, region I–VII [6]. Region II is a cysteine-rich region responsible for the interaction between EBA-175 and GYPA on the erythrocyte surface [7]. Previous studies have shown a significant excess of nonsynonymous substitutions over synonymous substitutions in this region in *P. falciparum* [8–11]; however, no studies have assessed to date the ratio of nonsynonymous to synonymous substitutions in other closely related *Plasmodium* species. Nonsynonymous substitution rates of host and pathogen are expected to increase synergistically as a consequence of the host-pathogen co-evolutionary arms race. When human and macaque genomes were compared, the *gypa* gene shows the highest number of nonsynonymous substitutions per nonsynonymous sites among 280 genes [10]. In addition, the rate of nonsynonymous substitutions in *eba*-*175* gene is also the highest among 10 genes compared between *P. falciparum* and *P. reichenowi* [10]. Thus, the high level of genetic polymorphisms shown by *eba*-*175* and *gypa* is likely to result from host–pathogen coevolution.

Amino acid substitutions in region II of *eba*-*175* and *gypa* seem to have been enhanced by diversifying selection. However, it remains unclear how human evolution has influenced *P. falciparum eba*-*175* genetic diversity. To elucidate this, in the present study, the regions II and III of *eba*-*175* from a Thai *P. falciparum* population were examined. When compared with *P. reichenowieba*-*175* sequence, *eba*-*175* alleles from this population can be traced back to approximately 130–140 thousand years ago. A further analysis for a large sequence dataset of *P. falciparumeba*-*175* alleles from across the world revealed an Asian-specific basal cluster in a phylogenetic tree. In addition, an evolutionary analysis detected a significant excess of nonsynonymous substitutions over synonymous substitutions in the region II of *eba*-*175* within the Thai *P. falciparum* population, but not within *Plasmodium* populations parasitizing African apes. Thus, the results from the present study suggest that human dispersal out of Africa contributed to the genetic diversification of *P. falciparum eba*-*175*.

## Methods

### Ethics statement

This study was approved by the Institutional Review Board of the Faculty of Tropical Medicine, Mahidol University, and the Research Ethics Committee of the School of Medicine, The University of Tokyo.

### Subjects

Peripheral blood samples were obtained from 203 *P. falciparum*-infected patients from Thailand. In addition, a dataset of nucleotide sequences including isolates of human *P. falciparum*, gorilla (*Gorilla gorilla*) *Plasmodium*, chimpanzee *Plasmodium*, and *P. reichenowi* was built from data obtained from [12].

### Experimental procedures for DNA sequencing of *eba*-*175*

Genomic DNA was extracted from pretreated peripheral blood samples from patients infected with *P. falciparum* using a QIAamp Blood Kit (Qiagen, Hilden, Germany). DNA fragments covering a part of the *eba*-*175* coding sequence (regions II and III) of *P. falciparum* were amplified by PCR using the following sets of primers: EBA175-fragment1, 5′-ggaagaaatacttcatctaataacg-3′ (forward) and 5′-catcctttacttctggacacatcg-3′ (reverse), and EBA175-fragment2, 5′-gagactctgaaggttgaatgcaa-3′ (forward) and 5′-aggtgtattagacatatcttggtc-3′ (reverse). These primers were designed based on the *eba*-*175* reference sequence from *P. falciparum* (GenBank accession no. X52524). PCR amplification was performed in a 13.0-µL reaction mixture containing 0.125 µL (0.125 µM) each of forward and reverse primers, 0.125 µL TAKARA LA Taq™ (5 units/µL), 1.25 µL 10× LA PCR™ Buffer II (Mg^2+^ free), 1.25 µL 25 mM MgCl_2_, 1.25 µL 2.5 mM dNTP mixture, 0.5 µL (5 ng) of genomic DNA template, and 8.375 µL dH_2_O using a GeneAmp^®^ PCR System 9700 (Applied Biosystems, Foster City, CA, USA). The PCR cycling conditions for each primer pair were 60 s initial denaturation at 94°C, followed by 40 cycles of 30 s denaturation at 94°C, 30 s annealing at 56°C, and 150 s extension at 72°C, and a final step of 5 min extension at 72°C. The PCR products were subsequently sequenced using an ABI Prism 3100 Genetic Analyzer (Applied Biosystems, Foster City, CA, USA). Primer sequences used for direct sequencing are available upon request. The isolates showing multiple superimposed electropherogram peaks at a single site following PCR-direct sequencing and a secondary peak greater than 30% of the predominant peak were considered to be mixed infections and excluded from further analyses. Low-quality sequences (i.e., high background noise or too weak signal) were also excluded. As a result, 131 sequences representing the single or most abundant sequence in each DNA sample were included in the analyses.

### Data analyses

The nucleotide sequences obtained were aligned and translated into putative amino acid sequences using MEGA v.5.2 [13]. To examine the phylogenetic relations among 32 distinct *eba*-*175* Thai *P. falciparum* alleles and two *eba*-*175P. reichenowi* alleles (CBXM000000000 and AJ251848), a maximum likelihood (ML) tree was constructed based on the Hasegawa–Kishino–Yano model [14]. To obtain the ML tree, a nearest-neighbor-interchange (NNI) search was applied. In addition, a neighbor-joining (NJ) [15] tree was generated using 194 *eba*-*175* partial region II sequences from *P. falciparum* worldwide and two *P. reichenowi* sequences (CBXM000000000 and AJ251848), based on the Nei–Gojobori (NG) model [16] and the Jukes–Cantor (JC) correction [17]. The construction of phylogenetic trees and estimation of best-fit substitution model for the ML tree were implemented in MEGA v.5.2 [13]. All the positions containing insertions/deletions were eliminated from the analyses (complete deletion option). Branch support values were computed by bootstrap analyses with 1,000 replications. A network of 194 *P. falciparum* and two *P. reichenowieba*-*175* alleles was also constructed based on synonymous substitutions using the neighbor-net method [18] in SplitsTree4 ver. 4.13.1 [19].

The time to the most recent common ancestor (tMRCA) of the *eba*-*175* alleles from Thai *falciparum* was estimated from the linearized tree based on synonymous substitutions among the 32 distinct *eba*-*175* alleles by using the MEGA v5.2 [13]. The neutral substitution rate was calculated assuming that *P. falciparum* and *P. reichenowi* diverged 6 million years ago (MYA) [5, 20]. In addition, the MCMCTree program in the PAML 4.8 package [21] was used to estimate tMRCA based on the amino acid sequences. The minimum and maximum age constraints on the root age (the divergence time between *P. falciparum* and *P. reichenowi*) were set to 5 and 7 MYA, respectively. The tMRCA estimation was based on a WAG model [22] for amino acid substitutions. In the MCMC process, sampling occurred every 100 generations for 10,000 generations and the first 50,000 generations were discarded as burn-in.

To detect the signatures of natural selection, the number of nonsynonymous substitutions per nonsynonymous site (*d*_N_) and synonymous substitutions per synonymous site (*d*_S_) for all the pairs formed by the 32 distinct Thai *P. falciparum* alleles, 16 from chimpanzee *Plasmodium* spp., 11 from gorilla *Plasmodium* spp., and four from *P. reichenowi* sequences were estimated using the NG model with the JC correction in MEGA v.5.2 [13]. Significant difference between *d*_N_ and *d*_S_ was assessed by Wilcoxon signed-rank test. For all the 131 Thai *P. falciparum* isolates, the numbers of nonsynonymous substitutions per nonsynonymous site (*π*_N_) and synonymous substitutions per synonymous site (*π*_S_) were also calculated in the same manner as *d*_N_ and *d*_S_. In addition, the McDonald–Kreitman (MK) test [23] was performed for detecting natural selection signal using DnaSP v5 software [24]. Tajima’s *D* test [25] was performed for 131 Thai *P. falciparumeba*-*175* sequences using DnaSP v5 software [24], where the test statistic was analytically calculated. A two-sided *P* value of less than 0.05 was considered statistically significant.

A Wu–Kabat plot was used to estimate the level of amino acid variability for the 32 distinct Thai *P. falciparum eba*-*175* alleles [26]. The Wu–Kabat plot estimates the level of variability for each amino acid position in the sequence alignment, measured as the number of amino acids at each site divided by the maximum frequency of amino acid for all sites.

## Results

### Detection of *eba*-*175* alleles

**Fig. 1:**
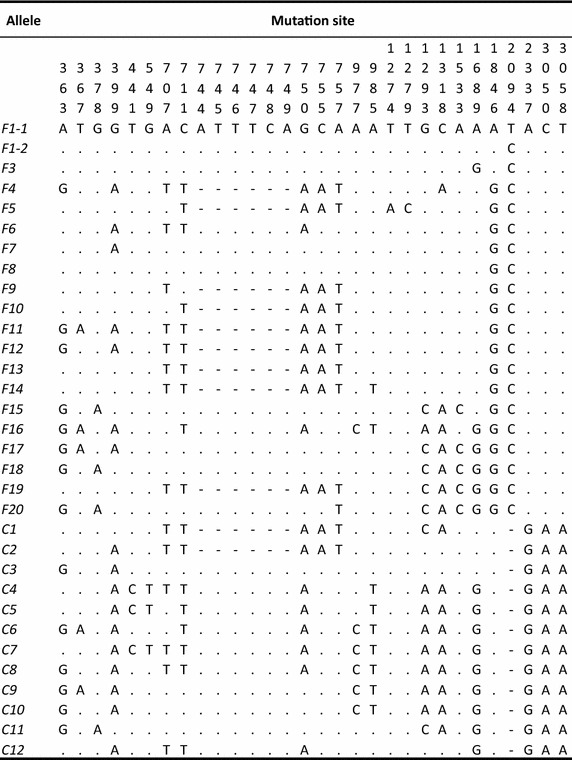
The 32 *eba*-*175* alleles detected from 131 Thai patients infected with malaria. *Dots* indicate identity with allele C1 nucleotide sequence. *Dashes* indicate insertions/deletions. F and C segments are not shown.

The nucleotide sequences of regions II and III of *P. falciparumeba*-*175* were obtained by PCR-direct sequencing. The *eba*-*175* region III showed highly divergent dimorphic segments, the F and C segments [termed *Fseg* (423 bp) and *Cseg* (342 bp)], as reported by Ware et al. [27]. A total of 32 distinct alleles [20 *Fseg* alleles (ca. 2,740 bp) and 12 *Cseg* alleles (ca. 2,660 bp)], defined by 30 polymorphic sites including insertions/deletions (site 744–749, and 2094), were detected from 131 *P. falciparum* isolates from Thailand [Fig. 1; *Fseg* alleles: *F1*_*1*–*F20* (Genbank accession numbers LC008232–LC008251) and *Cseg* alleles: *C1*–*C12* (Genbank accession numbers LC008252–LC008263)]. The nucleotide sequences of the 32 alleles were translated into 31 distinct amino acid sequences. Synonymous substitutions were found at only two sites in the allele sequences. On the other hand, nonsynonymous substitutions were found at 21 sites, not including an insertion/deletion site. The F and C segments were excluded from further analyses.

### Phylogenetic relations among *eba*-*175* alleles

**Fig. 2:**
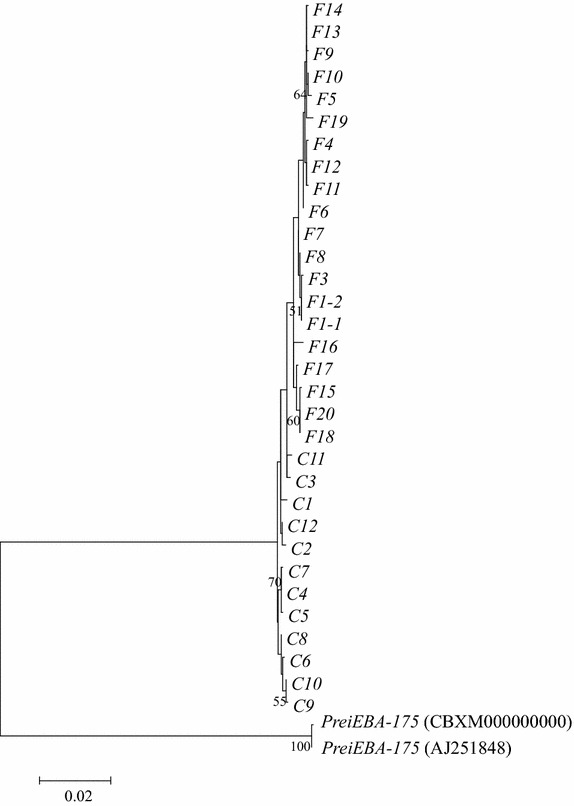
Maximum likelihood tree of *eba*-*175* (2,304 bp) regions II and III from Thai *Plasmodium falciparum*. The distance based on nucleotide substitutions was calculated by the Hasegawa–Kishino–Yano model. Two *P. reichenowi* *eba*-*175*-like (*Prei_eba*-*175*) sequences were used as outgroups. Only bootstrap values larger than 50% are shown.

**Fig. 3:**
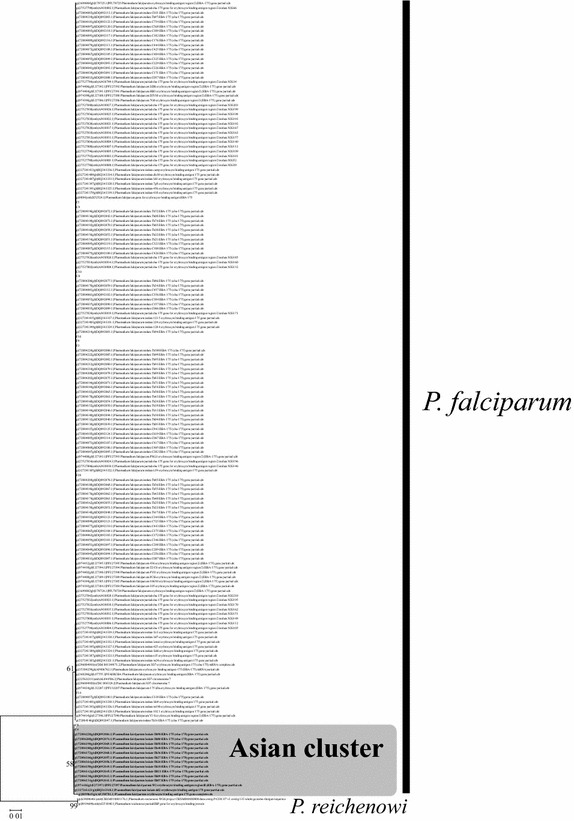
Neighbor-joining tree of *eba*-*175* partial region II (1,692 bp) of *P. falciparum* across the world. The distance based on synonymous substitutions was calculated using the Nei–Gojobori method with Jukes–Cantor model. Two *P. reichenowi* *eba*-*175*-like sequences were used as outgroups. Only bootstrap values larger than 50% are shown.

A ML tree was constructed based on the nucleotide sequences of 32 *eba*-*175* alleles from Thai *P. falciparum,* and two *eba*-*175* alleles from *P. reichenowi* (Fig. 2). In this ML tree, *Fseg* alleles formed a monophyletic clade with a relatively low bootstrap value (51%). A NJ tree was generated based on synonymous substitutions in region II to further analyze the phylogenetic relations among 194 *P. falciparum eba*-*175* alleles, including a large sequence dataset from Genbank [12] (Fig. 3). In particular, in this NJ tree, some *P. falciparum* alleles isolated from Asia formed a single clade rooted in the most basal node from where all *P.falciparum* sequences diverged. All the alleles included in the Asian clade were characterized by a mutation in site 441, as shown in Fig. 1 (Asian-specific alleles contain cytosine nucleotide at site 441, where all other sequences contain a thymine). This Asian-specific cluster was also supported by the neighbor-net method (Additional file 1) and suggests that an ancestral allele in the cluster emerged in Asia and then rapidly spread across Asia after the out-of-Africa.

### Estimated divergence time

The *d*_s_ values for regions II and III for all the pairs formed by the 32 distinct *eba*-*175* alleles were calculated to estimate tMRCA of *eba*-*175* alleles in the Thai population. The neutral mutation rate of the *eba*-*175* gene was extrapolated from the calibration point derived from the divergence time of *P. falciparum* and *P. reichenowi* (6 MYA) [5, 20] by using the linearized tree of *eba*-*175* alleles in the MEGA program [13]. Thus, the estimated neutral mutation rate was 1.0 × 10^−8^ per site per year. Accordingly, the estimated tMRCA for *eba*-*175* was approximately 0.14 MYA. The number of synonymous substitutions observed in Thai *P. falciparum* isolates was small (maximum *d*_S_ value = 2); consequently, MCMCTree was used to estimate the tMRCA of *eba*-*175* alleles based on amino acid substitutions within region III. In this case, the estimated tMRCA was approximately 0.13 MYA (95% highest posterior density confidential interval = 0.07–0.23 MYA). Notably, these estimations rely on the assumption that *P. falciparum* and *P. reichenowi* diverged 6 MYA.

### Diversifying selection on *eba*-*175* region II

To search a signal of natural selection on region II of *eba*-*175* in Thai *P. falciparum*, the *d*_N_ and *d*_S_ values for 32 long sequences (2,310 bp) were calculated (Table 1). A comparison of the two values revealed that the *d*_N_ was significantly larger than *d*_S_ (*P* value <2.2 × 10^−16^, Wilcoxon signed-rank test), suggesting that diversifying selection has influenced *eba*-*175* diversification in human *P. falciparum*. In addition, this result was further supported by the comparison between *π*_N_ and *π*_S_, including the allele frequencies of 131 Thai *P. falciparum* isolates (*π*_N_/*π*_S_ = 2.62; *P* value <2.2 × 10^−16^, Wilcoxon signed-rank test). Subsequently, it was examined whether diversifying selection has also operated within other taxa, using sequence data from 16 and 11 *Plasmodium* spp. infecting chimpanzees and gorillas, respectively, and four *P. reichenowi* isolates (Table 1). Long sequence data from African apes *Plasmodium* isolates were unavailable; consequently, region II short sequences (390–396 bp) were used for this analysis. Similar to the results using long sequences, *d*_N_ was significantly larger than *d*_S_ in human *P. falciparum* (*P* value <2.2 × 10^−16^, Wilcoxon signed-rank test); on the other hand, *d*_N_ was significantly smaller than *d*_S_ in African apes *Plasmodium* (each *P* value <2.2 × 10^−16^, Wilcoxon signed-rank test for chimpanzee and gorilla *Plasmodium* isolates). No significant differences between *d*_N_ and *d*_S_ were detected for *P. reichenowi* (*P* value = 0.32, Wilcoxon signed-rank test). These results suggest that diversifying selection has only acted on *eba*-*175* in human *P. falciparum*.

**Table 1:** Comparison of nonsynonymous (*d*_N_) and synonymous (*d*_S_ substitution rates in *Plasmodium eba-175* region II)

Species	*N*	Length (bp)	*d*_N_	*d*_S_	*d*_N_/*d*_S_	*P* value
Human *Plasmodium falciparum* isolates	32	2,310	0.005 ± 0.001	0.002 ± 0.001	2.98	<2.2 × 10^−16^
	32	390	0.010 ± 0.004	0.000 ± 0.000	N/A	N/A
Chimpanzee *Plasmodium* isolates	16	396	0.089 ± 0.013	0.110 ± 0.026	0.81	<2.2 × 10^−16^
Gorilla *Plasmodium* isolates	11	396	0.169 ± 0.019	0.207 ± 0.037	0.82	<2.2 × 10^−16^
*Plasmodium reichenowi*	5	393	0.003 ± 0.002	0.005 ± 0.005	0.47	0.32

*N/A* not applicable.

The McDonald–Kreitman (MK) test [23] for sequences of *eba*-*175* region II in 32 Thai *P. falciparum* alleles and one in *P. reichenowi* showed that the ratio of nonsynonymous to synonymous polymorphic sites within species (*P*n/*P*s = 19/1) was significantly higher than that of fixed sites between species (*D*n/*D*s = 116/48; *P* = 0.017, Fisher’s exact test). However, the MK test for *Plasmodium* isolates from African apes did not show significant differences between *P*n/*P*s and *D*n/*D*s (*P*n/*P*s = 59/23 and *D*n/*D*s = 14/6, *P*-value = 1.00 for 16 chimpanzee *Plasmodium* isolates and one *P. reichenowi*; *P*n/*P*s = 81/29 and *D*n/*D*s = 6/4, *P* = 0.46 for 11 gorilla *Plasmodium* isolates and one *P. reichenowi*). The results from the MK test suggest that either purifying selection or diversifying selection has acted on *eba*-*175* in Thai *P. falciparum* population.

Tajima’s *D* test [25] was used to test for departure from selective neutrality in 131 *eba*-*175* sequences of Thai *P. falciparum*. This test compares two population genetic parameters, one estimated from mean pairwise nucleotide differences and the other from the number of mutations. The results revealed a significant positive Tajima’s *D* value (Tajima’s *D* statistic = 3.01 and *P* value <0.01), suggesting the balancing selection on *eba*-*175* in Thai *P. falciparum*. The possibility of a recent reduction in population size that provided the significant positive Tajima’s *D* statistic may be excluded, since both the *d*_N_/*d*_S_ ratio test and the MK test also suggested the existence of positive diversifying selection.

To assess the relation between amino acid variability and the erythrocyte binding site, the level of amino acid variability at each site was examined using a Wu–Kabat plot (Additional file 2). The Wu–Kabat plot showed that the level of amino acid variability at some codon sites outside the sites of direct interaction between *P. falciparum* EBA-175 and human GYPA molecules [28] was higher than the variation level at their interaction sites. These variable sites may be located in regions recognized by human antibodies if the higher degree of variation is caused by diversifying selection favoring mutations to other amino acids.

## Discussion

Modern humans are believed to have emerged in Africa approximately 0.2 MYA and subsequently dispersed and colonized other continents after their exodus from Africa (known as the “out-of-Africa event”) approximately 0.1 MYA, although the accuracy of these estimates remains a contentious issue [29–32]. Here tMRCA for Thai *P. falciparumeba*-*175* was examined, and our results, 0.14 MYA from synonymous substitutions and 0.13 MYA from amino acid substitutions, suggest that the tMRCA of Thai *P. falciparum* may predate the out-of-Africa event but after the emergence of modern humans. However, this estimation relies largely on the assumption that *P. falciparum* diverged from *P. reichenowi* 6 MYA [5, 20]. A recent phylogenetic analysis for *Plasmodium* infecting Homininae species suggested that *Plasmodium* infecting gorillas was transmitted to modern humans in recent times [33]. This possible recent evolutionary origin of human *P. falciparum* is incompatible with the present assumption of that *P. falciparum/P. reichenowi* divergence time is similar to that of humans/chimpanzees (i.e., ca. 6 MYA [34–37]). However, regardless of the tMRCA estimate, ancestral human dispersal and recent increase in human population size, resulting in the increase in *P. falciparum* population size, are likely to have contributed to the *eba*-*175* diversification of *P. falciparum* (i.e., the emergence of Asian-specific clade).

The comparison of *d*_N_/*d*_S_ ratios of *eba*-*175* region II sequences among *Plasmodium* species showed an excess of nonsynonymous substitutions over synonymous substitutions in human *P. falciparum* but not in *Plasmodium* spp. infecting African apes and *P. reichenowi*. The result suggests that diversifying selection has affected *eba*-*175* region II in Thai *P. falciparum*, as previously reported [8–11]. The results on *π*_N_/*π*_S_ ratios and MK test [23] also supported the diversifying selection hypothesis. On the other hand, the estimated *d*_N_/*d*_S_ ratios and MK test [23] suggested that *eba*-*175* genetic diversities in other *Plasmodium* relatives have not been affected by diversifying selection. Thus, the EBA-175 protein may be the target for the immune response against *P. falciparum* malaria only in humans.

In this study, the tMRCA was estimated under the assumption of no inter-allelic recombination. The presence of inter-allelic recombination may affect the estimation of tMRCA. Since the DnaSP program [24] suggested the existence of recombination among 32 *eba*-175 alleles (Minimum number of recombination events, Rm: 8), we investigated the possible recombinants by using the GENECONV program [38], and detected 21 possible recombinants. After the removal of 21 possible recombinants, we recalculated the tMRCA of the remaining 11 *eba*-*175* alleles in Thai *P. falciparum*. The estimated tMRCA was approximately 0.12 MYA that was roughly equivalent to the tMRCA (0.14 MYA) estimated by using all of 32 alleles. In addition, the *d*_N_/*d*_S_ ratio of 11 sequences not including recombinant sequences (2.46) was not largely different from that of 32 sequences (2.98). Thus, our results seem not to be affected by the presence of possible recombinants.

## Conclusions

The genetic diversity in region II of *P. falciparumeba*-*175* seems to have been increased after the exodus from Africa of ancestral modern humans. As a consequence of the human expansion followed by an increase in *P. falciparum* population size, diversifying selection may have efficiently maintained the nonsynonymous substitutions in region II of Thai *P. falciparumeba*-*175*. Human dispersal out of Africa would have had a major impact on molecular evolution of *P. falciparumeba*-*175*.

## Additional files

Additional file 1: Neighbor-net network of *eba*-*175* alleles from a varied populations worldwide. The network was constructed based on synonymous substitutions of the partial region II from *Plasmodium falciparum*. Two *P. reichenowi* *eba*-*175* sequences were used as outgroups. Additional file 2: Level of variability for amino acid residues among *eba*-*175* alleles estimated using a Wu-Kabat plot. Amino acid sequences from regions II and III of *eba*-*175* alleles were used to construct a Wu–Kabat plot. The ordinate axis represents the level of amino acid variability. The abscissa axis represents the amino acid position. Pink bars indicate residues involved in the interaction with human GYPA molecules [28].
