# Supplementary material for: Genetic evidence for contribution of human dispersal to the genetic diversity of EBA-175 in Plasmodium falciparum
Source: Malar J. 2015 Aug 1;14:293. doi: 10.1186/s12936-015-0820-2 (PMC4522064; doi:10.1186/s12936-015-0820-2)
Supplement: Additional file 1: — Neighbor-net network of eba-175 alleles from a varied populations worldwide. The network was constructed based on synonymous substitutions of the partial region II from Plasmodium falciparum. Two P. reichenowi eba-175 sequences were used as outgroups. [file 12936_2015_820_MOESM1_ESM.pdf]

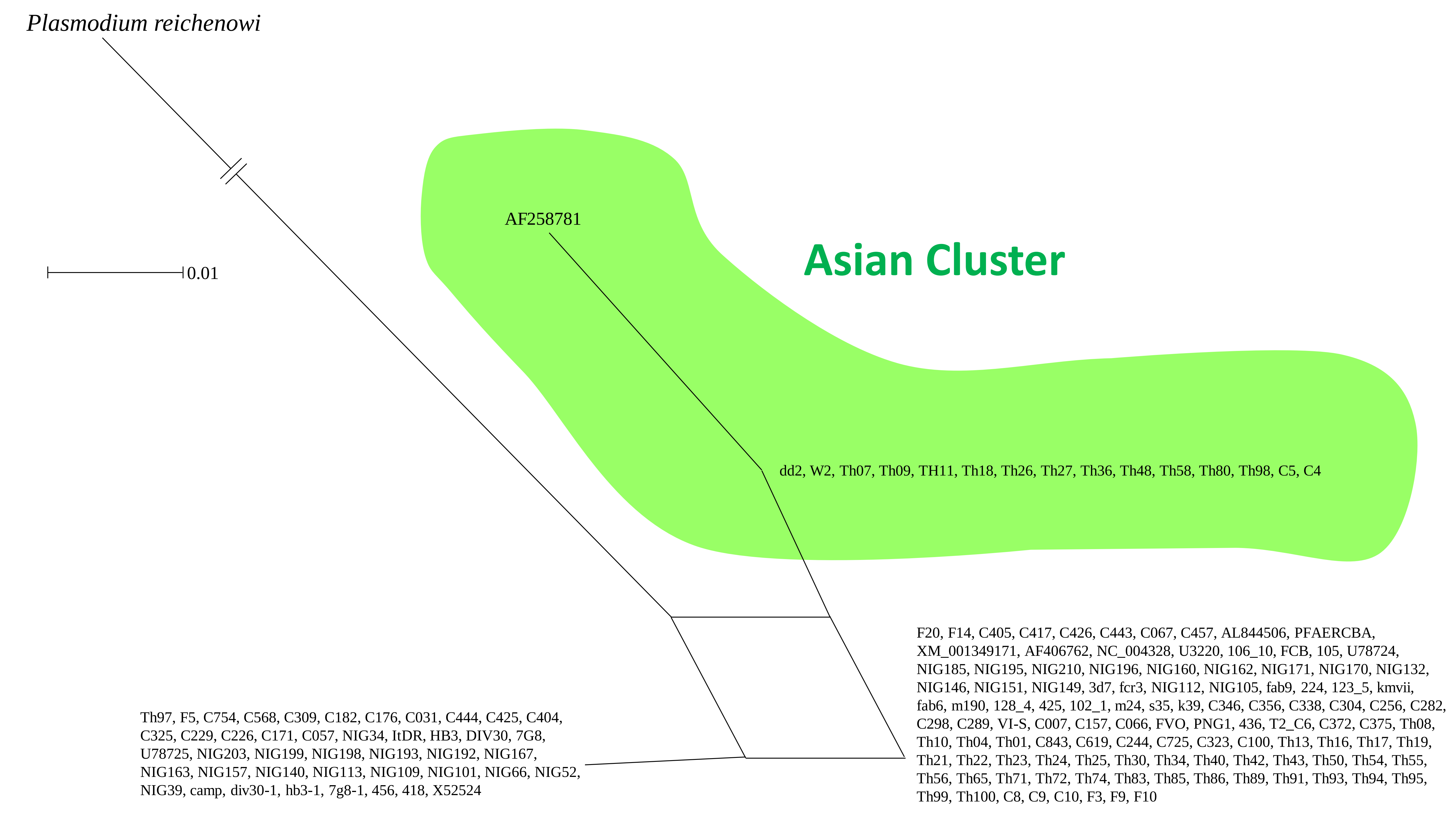
Plasmodium reichenowi
AF258781
0.01
Asian Cluster
dd2, W2, Th07, Th09, TH11, Th18, Th26, Th27, Th36, Th48, Th58, Th80, Th98, C5, C4
F20, F14, C405, C417, C426, C443, C067, C457, AL844506, PFAERCBA, XM_001349171, AF406762, NC_004328, U3220, 106_10, FCB, 105, U78724, NIG185, NIG195, NIG210, NIG196, NIG160, NIG162, NIG171, NIG170, NIG132, NIG146, NIG151, NIG149, 3d7, fcr3, NIG112, NIG105, fab9, 224, 123_5, kmvii, fab6, m190, 128_4, 425, 102_1, m24, s35, k39, C346, C356, C338, C304, C256, C282, C298, C289, VI-S, C007, C157, C066, FVO, PNG1, 436, T2_C6, C372, C375, Th08, Th10, Th04, Th01, C843, C619, C244, C725, C323, C100, Th13, Th16, Th17, Th19, Th21, Th22, Th23, Th24, Th25, Th30, Th34, Th40, Th42, Th43, Th50, Th54, Th55, Th56, Th65, Th71, Th72, Th74, Th83, Th85, Th86, Th89, Th91, Th93, Th94, Th95, Th99, Th100, C8, C9, C10, F3, F9, F10
Th97, F5, C754, C568, C309, C182, C176, C031, C444, C425, C404, C325, C229, C226, C171, C057, NIG34, ItDR, HB3, DIV30, 7G8, U78725, NIG203, NIG199, NIG198, NIG193, NIG192, NIG167, NIG163, NIG157, NIG140, NIG113, NIG109, NIG101, NIG66, NIG52, NIG39, camp, div30-1, hb3-1, 7g8-1, 456, 418, X52524
