# Supplementary figures and images for: Genetic evidence for contribution of human dispersal to the genetic diversity of EBA-175 in Plasmodium falciparum
Source: Malar J. 2015 Aug 1;14:293. doi: 10.1186/s12936-015-0820-2 (PMC4522064; doi:10.1186/s12936-015-0820-2)

Amino acid variability

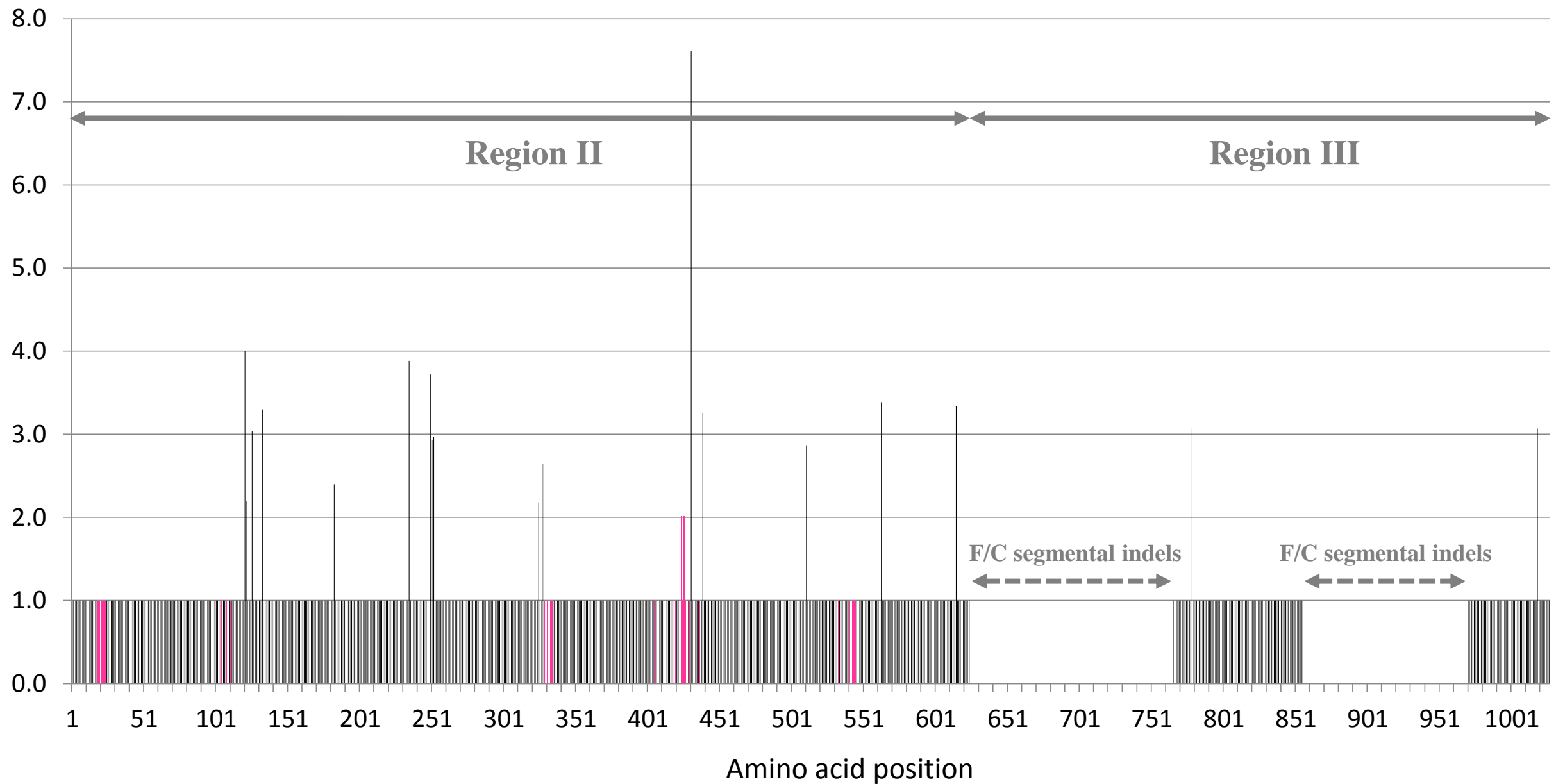

Supplement: Additional file 2: — Level of variability for amino acid residues among eba-175 alleles estimated using a Wu-Kabat plot. Amino acid sequences from regions II and III of eba-175 alleles were used to construct a Wu–Kabat plot. The ordinate axis represents the level of amino acid variability. The abscissa axis represents the amino acid position. Pink bars indicate residues involved in the interaction with human GYPA molecules [28]. [file 12936_2015_820_MOESM2_ESM.pdf]
